# Changes of individual choroidal thickness post-uneventful cataract surgery determined by spectral-domain optical coherence tomography over a 3-month period

**DOI:** 10.3389/fmed.2026.1750805

**Published:** 2026-02-18

**Authors:** T. Peschaut, R. Riedl, L. Harling, J. Gran, L. Posch-Pertl, W. M. Glatz, T. Falb, D. Djavid, D. Ivastinovic, A. Wedrich, E. M. Malle, M. Großpötzl

**Affiliations:** 1 Department of Ophthalmology, Medical University of Graz, Graz, Styria, Austria; 2 Institute for Medical Informatics, Statistics and Documentation, Medical University of Graz, Graz, Styria, Austria; 3 Department of Ophthalmology, Faculty of Medicine, University of Rijeka, Rijeka, Croatia

**Keywords:** cataract, choroid, phacoemulsification, postoperative, retina, thickness, volume

## Abstract

**Purpose:**

To evaluate changes in choroidal thickness and choroidal volume after uncomplicated cataract surgery using enhanced depth imaging optical coherence tomography (EDI-OCT) over a 3-month period.

**Methods:**

For this retrospective analysis, data collected in a different, prospective study previously conducted at the Department of Ophthalmology, Medical University of Graz, Austria, was utilized and further analysed. In the original study, retinal layer thickness was measured using spectral-domain optical coherence tomography (SD-OCT) before surgery and at 1 day, 1 month, and 3 months postoperatively. During the current study, the automated segmentation of the retina was manually adjusted to the choroid, using EDI-OCT. Subsequently, choroidal thickness in the central subfield (1 mm) and in the superior, inferior, temporal and nasal sectors of the inner ring (3 mm) as well as choroidal volume of the inner and outer ring (3 mm and 6 mm) of the “early treatment diabetic retinopathy study” (ETDRS) grid were extracted. We included 41 patients who underwent uncomplicated cataract surgery between February 2016 and October 2017. Patients with pre-existing ocular or systemic conditions, intraoperative complications or insufficient image quality were excluded.

**Results:**

A total of 42 eyes of 41 patients (mean age 70.2 ± 8.7 years; 51.2% male) were analysed. In almost all ETDRs sectors, a significant increase in choroidal thickness and volume with a maximum at 1 month postoperatively was found (*p* ≤ 0.05), followed by a decrease by month three, without returning to baseline levels. Five eyes (11.9%) developed cystoid macular edema. The postoperative pattern of structural changes was comparable in patients with and without cystoid macular edema.

**Conclusion:**

Our results suggest that there are significant changes in choroidal thickness and volume after uncomplicated cataract surgery. The most probable causes are acute inflammatory processes, hemodynamic adjustments to postoperative intraocular pressure fluctuations and metabolic reactions to improved optical transmission.

## Introduction

Cataract surgery is one of the most performed ophthalmic procedures worldwide, with phacoemulsification being the standard technique due to its efficacy and safety. While the primary goal of cataract surgery is to restore visual acuity by removing the opacified lens, recent studies have highlighted its potential impact on ocular structures, including changes in retinal thickness (RT) and choroidal thickness (CT) (1–4).

Cystoid macular edema (CME) is a possible postoperative complication following uncomplicated cataract surgery and represents a leading cause of reduced visual acuity in the early postoperative period; however, in most cases, it is self-limiting (5). Advances in imaging technologies, such as spectral-domain optical coherence tomography (SD-OCT) and enhanced depth imaging optical coherence tomography (EDI OCT), have enabled precise measurement of both RT and CT, facilitating the investigation of postoperative changes (6, 7).

Several studies have reported alterations in RT and CT after cataract surgery, though findings remain inconsistent. Previous studies demonstrated significant postoperative alterations in RT following uncomplicated cataract surgery while Yilmaz et al. and Akcam et al. focused on CT and found no significant or only transient alterations (1–4). Conversely, other studies reported a significant and persistent increase in subfoveal CT postoperatively, highlighting the need for further research to clarify these discrepancies (8, 9).

The mechanisms underlying these changes remain poorly understood, but potential factors include alterations in intraocular pressure, inflammation, and oxidative stress induced by the surgical procedure (1–3, 10). Additionally, variations in study design, patient demographics, and imaging techniques may contribute to the varying results observed in literature. Understanding the impact of cataract surgery on both RT and CT is clinically relevant, as it may influence postoperative visual outcomes and the management of coexisting retinal pathologies.

This study aims to evaluate changes in CT and choroidal volume (CV) following uncomplicated cataract surgery using SD-OCT with EDI to improve analysis of the choroid. By providing a comprehensive analysis of choroidal morphology over a defined period, this study seeks to contribute to the growing body of evidence on the subject and elucidate potential implications for clinical practice.

## Methods

For this retrospective analysis, we utilized and further analysed data previously collected in a different, prospective study at the Department of Ophthalmology, Medical University of Graz, Austria (1). The study was performed in accordance with the guidelines of the Declaration of Helsinki (11), including current revisions and was approved by the Ethics Committee of the Medical University of Graz (Number 1191/2025).

During the original study, RT was measured using SD-OCT before surgery and at 1 day, 1 month, and 3 months postoperatively, utilizing the device’s built-in follow-up mode. Scans were centered on the anatomical fovea using the Heidelberg Spectralis™ device (HEYEX version 2.6.4, Heidelberg Engineering, Germany). The analysis focused on individual retinal layers in the central subfield and inner ring of the “early treatment diabetic retinopathy study” (ETDRS) grid. SD-OCT scans comprised of 31 B-scans at a wavelength of 825 nm. The images had a resolution of 7 μm axially × 14 μm laterally, and an inter-scan spacing of 240 μm. EDI was applied to optimize visualization and analysis of the choroid.

**Figure 1:**
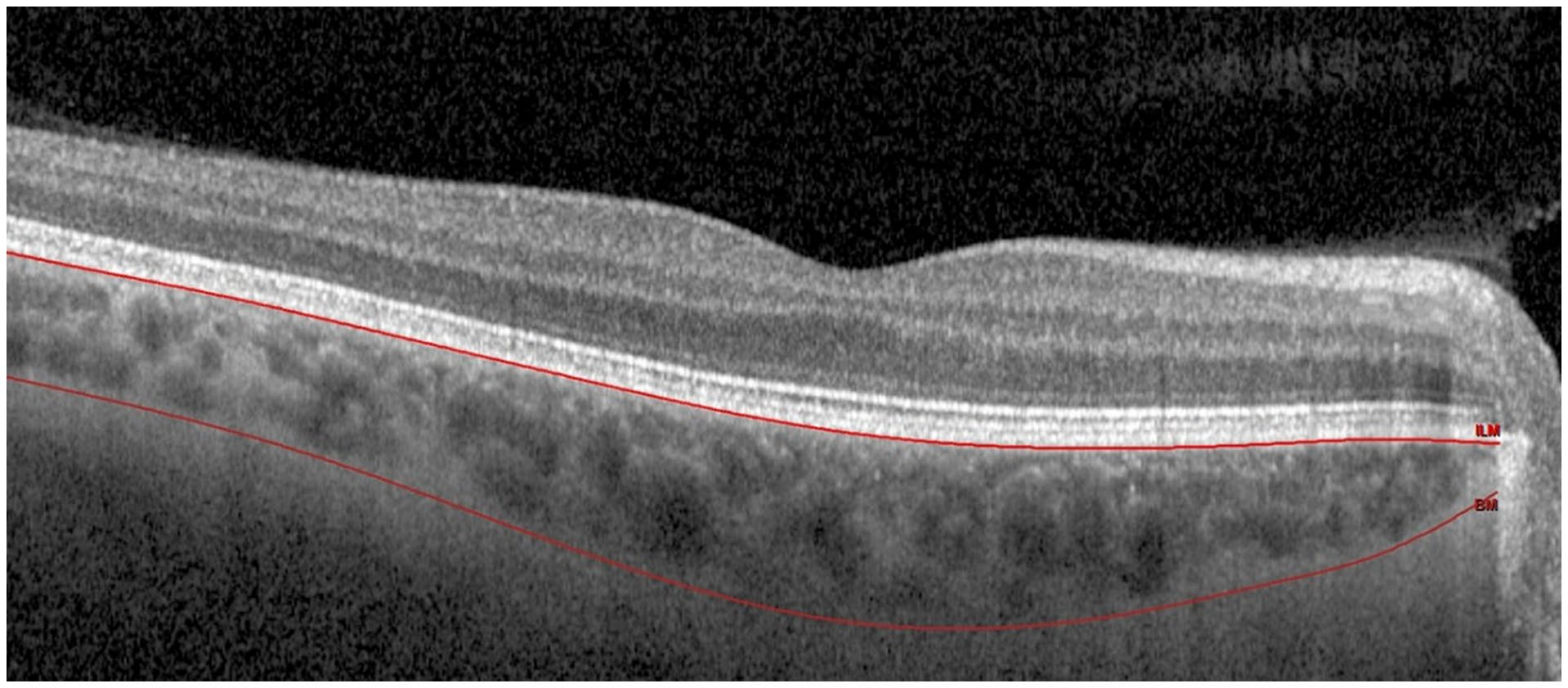
Boundaries of the choroid on enhanced depth imaging optical coherence tomography. The segmentation lines of the inner limiting membrane (ILM) and Bruch’s membrane (BM) were moved accordingly to facilitate the measurement of choroidal thickness and volume in the respective segments.

To enable retrospective analysis of CT, previously collected OCT data from the original study were re-evaluated. The automated segmentation of the retinal layers was manually adjusted to the choroid using the segmentation tool integrated in the Spectralis software by individuals TP and LH (Figure 1). In the preprogrammed analysis mode, HEYEX automatically calculates retinal thickness and volume between the inner limiting membrane (ILM) and Bruch’s membrane (BM). Since automated segmentation of the choroid is not yet available in the software, the ILM and BM segmentation lines were manually adjusted to align with the visible boundaries of the choroid. This allowed the software to compute the choroidal thickness and volume in the defined ETDRS subfields, providing quantitative assessment of choroidal structural changes across all follow-up time points. Subsequently, the following values were extracted from the ETDRS grid: CV of the inner ring (=3 mm) and outer ring (=6 mm), CT of the superior, inferior, temporal and nasal inner rings (=3 mm) and CT of the central subfield (=1 mm). Data from 42 eyes of 41 patients who underwent uneventful cataract surgery between February 2016, and October 2017 were included in this analysis. Only data from the operated eyes were included. In one case, the operation was performed in both eyes of the same patient, data of both eyes was included.

Cystoid macular edema (CME) was defined as any area of reduced reflectivity within the neurosensory retina, as detected by SD-OCT. For a more detailed analysis of both subclinical and clinical progression, patients presenting with CME (*n* = 5) were excluded from a subsequent subgroup analysis. No postoperative complications, such as elevated intraocular pressure, pronounced fibrin reaction, or endophthalmitis, were observed during the follow-up period.

Inclusion criteria comprised patients who underwent uncomplicated phacoemulsification cataract surgery with in-the-bag intraocular lens (IOL) implantation and had high-quality SD-OCT scans available at baseline and postoperative follow-up visits. Exclusion criteria included myopia greater than 5 diopters, the presence of any macular pathology (e.g., drusen, age-related macular degeneration, diabetic retinopathy, or retinal vascular occlusion), history of ocular inflammation (e.g., uveitis), glaucoma, ocular trauma, or previous intraocular surgery. Patients with systemic diseases, such as diabetes mellitus or systemic inflammatory conditions, as well as those receiving ongoing topical or systemic anti-inflammatory or anti-glaucoma medications, were also excluded. Further exclusion criteria were intraoperative complications (e.g., posterior capsule rupture, vitreous loss, or iris prolapse) and SD-OCT scans of insufficient quality due to media opacities or poor visibility of the choroid.

Cataract surgery was performed using standard phacoemulsification techniques with IOL implantation in the capsular bag. At the end of each procedure, 1 mg of intracameral cefuroxime (Curocef®, Glaxo Wellcome Pharma, Vienna, Austria) was administered. All surgeries were performed by one of 13 experienced cataract surgeons, each of whom had completed a minimum of 323 cataract procedures prior to the respective case. Postoperative treatment consisted of topical neomycin and betamethasone eye drops (Betnesol N®, TubiluxPharma, Rome, Italy) applied five times daily in a tapered regimen over 5 weeks, along with a dexamethasone and gentamycin ointment (Dexagenta®, Pharma GmbH, Leobendorf, Austria) used once daily at night for 1 week.

## Statistical analysis

Descriptive results are presented as mean ± standard deviation for continuous variables and as n and percent for categorical variables. To investigate changes in CT after uncomplicated cataract surgery, mixed model repeated measure (MMRM) models were used for the investigated layers separately. Visit (in categories preoperative, day 1, month 1 and months 3 postoperative), pre-operative best corrected visual acuity, lens status of the fellow eye (phakic and pseudophakic), duration of surgery and patient age were included as fixed effects. Accounting for the data dependencies, a random intercept for patient was included and an autoregressive AR (1) covariance structure for the visits was modelled. Overall significance for visits was tested by type 3 tests. In addition, least square means (LSMs) at the single visits were compared with each other and presented with LSM-differences (LSMDs) and their corresponding 95% confidence intervals (CIs). For the comparisons of the changes between the visits, Bonferroni correction was done. No imputation for missing data was applied. All analyses were performed in the whole cohort (*n* = 42 eyes), and in the sub-cohort without CME cases (*n* = 37 eyes). For statistical analysis, SAS 9.4 (SAS Institute, Cary NC) was used.

## Results

In total, 42 eyes of 41 patients were included in the evaluation, including 24 right eyes (57.1%) and 18 left eyes (42.9%). Of the respective contralateral eyes, 27 (64.3%) were phakic and 15 (35.7%) were pseudophakic. The average age of the included patients was 70.2 ± 8.7 years. 21 patients (51.2%) were male, 20 (48.8%) were female. The mean overall surgical duration was 14.9 min. As a result of missed postoperative follow-up appointments (*n* = 9) and insufficient image quality (*n* = 2) of the OCT examination, a total of 31 EDI-OCT scans were available for analysis on the first postoperative day, while adequate data of all 42 eyes was collected at the end of the first month. Due to insufficient image quality, only 37 scans were suitable for analysis at the end of the third month. In 5 eyes (11.9%), CME was detected 1 month after cataract surgery.

In all evaluated ETDRS sectors, a significant postoperative increase in CT was observed, most pronounced at 1 month following surgery. Although a subsequent decrease was noted at the 3-month follow-up, thickness values remained elevated compared to baseline. These changes were statistically significant across all sectors. The most marked increase occurred in the superior sector, rising from 244.76 ± 72.53 μm preoperatively to 259.64 ± 72.29 μm at 1 month postoperatively. At 3 months, a decline of thickness in the superior sector was observed, though values did not return to baseline levels.

CV followed a similar pattern, with a significant increase over the course of the study, peaking at 1 month postoperatively and showing a reduction thereafter. Nonetheless, CV remained above preoperative levels at 3 months as well. A decrease in both CT and CV was noted in all sectors on the first postoperative day (Table 1).

**Table 1:** Mean choroidal thickness [μm] and mean choroidal volume [mm^3^] throughout the visits (42 eyes of 41 patients), adjusted for patient age, best corrected visual acuity, lens status of the fellow eye (phakic and pseudophakic) and duration of surgery.

Parameter	ETDRS grid	Baseline values of the mean thickness/volume [μm/mm^3^] ± standard deviation				*p*-value (Typ 3 test MMRM)
		Preoperative *N* = 42	Day 1 postoperative *N* = 31	Month 1 postoperative *N* = 42	Month 3 postoperative *N* = 37	
CT	Central	231.24 (66.74)	226.13 (57.63)	243.90 (66.55)	241.24 (68.07)	**0.0021**
	Inferior	224.00 (63.98)	217.10 (57.55)	233.86 (64.87)	226.95 (65.30)	**0.0003**
	Nasal	215.36 (63.83)	208.45 (56.05)	227.62 (62.19)	221.05 (61.93)	**0.0011**
	Superior	244.76 (72.53)	236.35 (62.47)	259.64 (72.29)	258.05 (74.52)	**<0.0001**
	Temporal	233.43 (68.14)	227.71 (60.18)	245.55 (71.49)	243.14 (72.90)	**0.0001**
CV	Inner ring (=3 mm)	1.62 (0.46)	1.58 (0.40)	1.71 (0.46)	1.68 (0.47)	**<0.0001**
	Outer ring (=6 mm)	6.14 (1.75)	6.00 (1.49)	6.46 (1.71)	6.34 (1.76)	**<0.0001**

CT, Choroidal Thickness, CV, Choroidal Volume, MMRM, mixed model repeated measure; Bold: Statistically significant differences (*p* < 0.05) Anatomical mapping (ETDRS grid): central = 1 mm, inferior = 3 mm, nasal = 3 mm, superior = 3 mm, temporal = 3 mm.

In nearly all sectors of the ETDRS grid, a significant postoperative increase in CT was observed, particularly when comparing preoperative baseline measurements with those taken at one and 3 months after surgery. The most pronounced changes were found in the superior sector, with a significant increase of 14.88 μm (95% CI: 6.28–23.48) at 1 month and 15.36 μm (95% CI: 6.32–24.41) at 3 months. A significant increase of 13.25 μm (95% CI: 3.47–23.03) was also observed between postoperative day 1 and month 3. In contrast, no statistically significant differences were detected between month 1 and month 3, or between baseline and day 1 postoperatively (Table 2).

**Table 2:** Investigation of alterations in the mean choroidal thickness [μm] and mean choroidal volume [mm^3^] throughout the visits adjusted for patient age, best corrected visual acuity, lens status of the fellow eye (phakic and pseudophakic) and duration of surgery (42 eyes in 41 patients).

Parameter	ETDRS grid	Presented are the least square mean differences and 95% confidence interval (Bonferroni corrected) in μm/mm^3^					
		Day 1—Pre OP	Month 1—Pre OP	Month 3—Pre OP	Month 1—Day 1	Month 3—Day 1	Month 3—Month 1
CT	Central	2.68 (−6.29, 11.66)	**12.67 (2.97, 22.36)**	**12.55 (1.88, 23.23)**	**9.98 (1.01, 18.96)**	9.87 (−0.91, 20.66)	−0.11 (−8.68, 8.46)
	Inferior	−2.06 (−10.71, 6.58)	**9.86 (2.29, 17.43)**	6.51 (−1.43, 14.44)	**11.92 (3.28, 20.56)**	8.57 (−0.16, 17.31)	−3.35 (−11.46, 4.76)
	Nasal	1.63 (−6.35, 9.6)	**12.26 (3.44, 21.08)**	8.96 (−0.88, 18.8)	**10.64 (2.67, 18.61)**	7.33 (−2.43, 17.1)	−3.3 (−10.94, 4.34)
	Superior	2.11 (−6.88, 11.11)	**14.88 (6.28, 23.48)**	**15.36 (6.32, 24.41)**	**12.77 (3.77, 21.76)**	**13.25 (3.47, 23.03)**	0.48 (−8, 8.96)
	Temporal	1.57 (−6.49, 9.62)	**12.12 (4.01, 20.22)**	**11.91 (3.27, 20.56)**	**10.55 (2.5, 18.6)**	**10.34 (1.21, 19.48)**	−0.21 (−7.83, 7.42)
CV	Inner ring (=3 mm)	0.01 (−0.04, 0.06)	**0.09 (0.03, 0.14)**	**0.08 (0.02, 0.13)**	**0.08 (0.03, 0.13)**	**0.07 (0.01, 0.13)**	−0.01 (−0.06, 0.04)
	Outer ring (=6 mm)	0.04 (−0.15, 0.23)	**0.28 (0.12, 0.45)**	**0.26 (0.09, 0.43)**	**0.25 (0.06, 0.43)**	**0.22 (0.03, 0.4)**	−0.03 (−0.21, 0.15)

CT, Choroidal Thickness, CV, Choroidal Volume; Bold: Statistically significant differences (*p* < 0.05) Anatomical mapping (ETDRS grid): central = 1 mm, inferior = 3 mm, nasal = 3 mm, superior = 3 mm, temporal = 3 mm.

CV also increased significantly postoperatively in both the inner (3 mm) and outer (6 mm) ETDRS rings. In the inner ring, a significant volume increase of 0.09 mm^3^ (95% CI: 0.03–0.14) was observed between baseline and 1 month postoperatively. Furthermore, most significant increases were noted between day 1 and month 1 (0.08 mm^3^, 95% CI: 0.03–0.13) as well as between baseline and month 3 (0.08 mm^3^, 95% CI: 0.02–0.13). A similar pattern was observed in the outer ring, with most significant volume increases of 0.28 mm^3^ (95% CI: 0.12–0.45) between baseline and month 1, and 0.26 mm^3^ (95% CI: 0.09–0.43) between baseline and month 3. The most substantial changes occurred between baseline and the first postoperative month, while no significant differences were observed between months 1 and 3 (Table 2), indicating a stabilization after 3 months.

A comparison between the entire study cohort and the subgroup without CME revealed very similar trends in postoperative choroidal changes (Table 3).

**Table 3:** Investigation of alterations in the mean choroidal thickness [μm] and mean choroidal volume [mm^3^] throughout the visits adjusted for patient age, best corrected visual acuity, lens status of the fellow eye (phakic and pseudophakic) and duration of surgery in subcohort with no CME (37 eyes in 36 patients).

Parameter	ETDRS grid	Presented are the least square mean differences and 95% confidence interval (Bonferroni corrected) in μm/mm^3^					
		Day 1—Pre OP	Month 1—Pre OP	Month 3—Pre OP	Month 1—Day 1	Month 3—Day 1	Month 3—Month 1
CT	Central	1.92 (−7.58, 11.43)	**12.3 (1.66, 22.94)**	**12.23 (0.18, 24.27)**	**10.37 (0.87, 19.87)**	10.3 (−1.54, 22.14)	−0.07 (−9.24, 9.1)
	Inferior	−4.93 (−14.28, 4.42)	7.84 (−0.23, 15.91)	6.1 (−2.43, 14.63)	**12.77 (3.42, 22.12)**	**11.04 (1.63, 20.45)**	−1.73 (−10.53, 7.06)
	Nasal	0.9 (−7.56, 9.37)	**11.76 (2.03, 21.49)**	9.07 (−2.12, 20.26)	**10.85 (2.39, 19.32)**	8.17 (−2.61, 18.94)	−2.69 (−10.89, 5.51)
	Superior	1.66 (−7.96, 11.29)	**14.86 (5.5, 24.23)**	**15.94 (5.99, 25.89)**	**13.2 (3.57, 22.83)**	**14.28 (3.58, 24.97)**	1.08 (−8.04, 10.2)
	Temporal	0.91 (−7.93, 9.75)	**12.03 (3.23, 20.82)**	**12.31 (2.9, 21.72)**	**11.12 (2.27, 19.96)**	**11.4 (1.39, 21.41)**	0.28 (−8.11, 8.68)
CV	Inner ring (=3 mm)	0 (−0.06, 0.06)	**0.08 (0.02, 0.14)**	**0.08 (0.02, 0.14)**	**0.08 (0.03, 0.14)**	**0.08 (0.01, 0.14)**	0 (−0.06, 0.05)
	Outer ring (=6 mm)	0.01 (−0.2, 0.22)	**0.27 (0.09, 0.45)**	**0.26 (0.08, 0.45)**	**0.26 (0.05, 0.47)**	**0.26 (0.05, 0.46)**	−0.01 (−0.2, 0.19)

CT, Choroidal Thickness; CV, Choroidal Volume; Bold: Statistically significant differences (*p* < 0.05) Anatomical mapping (ETDRS grid): central = 1 mm, inferior = 3 mm, nasal = 3 mm, superior = 3 mm, temporal = 3 mm.

Both groups showed a significant increase in CT and CV, particularly within the first month after surgery, with the most pronounced changes observed in the superior sector. The overall pattern of early postoperative thickening followed by stabilization towards the third month was consistent across both cohorts, indicating that the presence of CME did not substantially alter choroidal structural changes. In both cohorts, CT and CV values did not return to baseline levels at the 3-month follow-up. There was no significant difference in the postoperative changes in CT and CV between female and male patients.

## Discussion

The results of our study show significant structural changes in CT and CV after uncomplicated cataract surgery, with the most pronounced increase after 1 month postoperatively. These observations largely coincide with the results of other studies describing similar early-stage choroidal reactions after phacoemulsification.

An increase in CT was documented in numerous studies within the first postoperative weeks to months, the most pronounced increase was usually recorded within the first four to 6 weeks—a pattern that was confirmed by our study (8–10, 12–15). In two studies, even after 6 months, CT was significantly increased (12, 14), while other authors report a decline in CT to baseline levels after just 1 month (4). In our analysis, we observed a decrease in CT and CV between 1 and 3 months postoperatively; however, the values did not return to their preoperative levels.

The documented postoperative changes in CT in the literature vary, Yilmaz et al. observed a slight, statistically insignificant increase in CT over 12 months, without returning to baseline levels (3), the cohort observed in the study by Ibrahim et al. showed a return of CT to nearly baseline levels after just 3 months (9). Wu et al. describe the most pronounced postoperative increase in CT at 6 months, negatively correlating with the measured intraocular pressure (IOP), which reached its minimum after 6 months postoperatively (14). Noda et al. report stabilized but still elevated CT after 6 months, the increase in CT was associated with a higher baseline CT and male sex (12). In our study, we found no significant difference in postoperative changes of CT and CV between female and male patients. A meta-analysis by Zeng et al. also showed an increase of CT without a return to baseline for at least 3 months postoperatively, the increase was greatest in Asian patients and in patients who did not receive postoperative therapy with nonsteroidal anti-inflammatory drugs (NSAIDs), no significant changes were found in patients with diabetes mellitus (13).

A separate analysis of CV is not frequently described in the literature. Whereas significant changes were observed during the follow-up period in our study, a prospective study by Pilotto et al. merely showed a statistically insignificant increase in CV after uncomplicated cataract surgery. In contrast to our study, this analysis included only nine eyes, with OCT imaging performed using a NIDEK device (Gamagori, Japan). OCT scans were analysed automatically by the device itself and were reviewed individually for accuracy. Postoperative treatment consisted of dexamethasone 0.15% four times daily for 1 week, combined with nepafenac 0.1% three times daily for 2 weeks, and thus differed from our postoperative regimen (16).

A different study by Reyhan et al. focused on the choroidal vascular index (CVI), i.e., the ratio of luminal to total choroidal area, and found no significant changes in CVI after phacoemulsification compared to scleral-fixed intraocular lens implantation (17). A study by İçöz including 50 eyes undergoing uneventful phacoemulsification evaluated structural and vascular changes in the choroid using EDI-OCT and CVI analysis. Subfoveal, nasal, and temporal choroidal thickness increased significantly at 1 month postoperatively but returned to baseline by 3 months. In contrast, the choroidal vascularity index (CVI) increased significantly at both one and 3 months compared to baseline, suggesting that phacoemulsification induces not only transient structural but also sustained vascular changes in the choroid. These findings support the potential utility of CVI in assessing choroidal vascular responses following cataract surgery (18).

The CVI was first introduced by Agarwal et al. in 2016 as a novel marker of choroidal vascularity and is calculated using a modified image binarization technique (19). In our study, “choroidal volume” (CV) provided a more feasible approach to quantify choroidal changes retrospectively using the available OCT data and the HEYEX software. While CV is also a relatively novel and less established marker, it allowed us to explore three-dimensional changes in the choroid across ETDRS subfields in a reproducible manner.

A recent overview of modern choroidal imaging highlights the challenge of accurately measuring CV, due to the choroid’s complex three-dimensional architecture, convoluted vascular arrangement, segmentation difficulties, lack of normative reference values and methodological variability across devices and algorithms (20).

In our analysis, we encountered 5 patients who developed CME 1 month after surgery, all of which subsided without further sequelae. Interestingly, measured changes in CT and CV were most pronounced at the 1-month follow-up. However, a sub-analysis and comparison of both the entire study cohort and the cohort without CME revealed no substantial difference in postoperative changes of CT and CV, indicating that the presence of CME did not substantially alter the overall trajectory of choroidal structural changes in our study.

The exact pathophysiological mechanisms behind the observed changes have not yet been conclusively clarified, but several factors seem to play a role. A postoperative inflammatory reaction is most frequently mentioned in the literature as the cause of the increase in CT and CV. Surgical trauma during phacoemulsification leads to the release of pro-inflammatory mediators such as prostaglandins and cytokines, which induce vasodilation, increased vascular permeability and interstitial fluid accumulation in the choroid (1–3, 9). Consequently, patients who receive postoperative anti-inflammatory therapy (e.g., in the form of local NSAIDs) show lower structural changes in retina and choroid (2, 13, 21–24). However, this hypothesis does not adequately explain long-term structural changes, since it can be assumed that the ocular inflammatory process will subside after a certain time. Another possible reason for a transient increase in CT and CV is the reduction of IOP after cataract surgery, which triggers compensatory hyperperfusion with reduced transmural pressure and venous outflow resistance in the choroid (3, 10, 14). A further possible factor triggering structural changes is the improved light transmission after removal of the opacified lens. The subsequent increase in retinal metabolic activity could result in a higher demand for choroidal perfusion and thus trigger vascular adjustment processes and an increase in CV (3, 12). This hypothesis could explain the persistent increase in CT and CV found in most cases, compared to inflammatory processes and fluctuations in IOP, which would seem to be factors of transient nature.

As mentioned above, evaluation of CV comparable to our analysis has only been conducted in one other study including a study population of 9 eyes, and accurate measurements remain challenging for a multitude of reasons (16, 20). Nevertheless, assessing CV after uncomplicated cataract surgery can provide valuable additional information beyond conventional thickness measurements. Volumetric analysis captures three-dimensional changes across the entire choroid, detecting subtle, widespread alterations that thickness measurements may miss, allowing for a more comprehensive assessment of the choroidal response to surgery, especially in the context of regional inflammatory or vascular changes that may not be uniformly distributed anatomically.

Furthermore, it is important to note that most of the aforementioned studies do not provide clear information on mean overall surgical duration, duration of phacoemulsification, or mean phacoemulsification energy. The measurements in our study were, among other factors, corrected for overall duration of surgery. These parameters are clinically relevant, as longer procedures and higher phaco energy may induce greater surgical trauma, heat production, and intraocular inflammatory response, all of which may directly influence CT, CV and postoperative outcomes. Without accounting for these variables, the interpretation and comparability of results across studies might be limited.

## Limitations

This study has several limitations: The retrospective design involves standardization and bias risks. The small, selected patient collective (only uncomplicated cataract operations, without relevant comorbidities) limits generalizability. The observation period of 3 months only allows statements on short-term changes. Manual segmentation of the choroid is personnel-dependent and error-prone, the term “CV” is not yet widely validated. The involvement of multiple surgeons may introduce inter-surgeon variability. However, all procedures were performed by experienced cataract surgeons, thereby minimizing potential surgeon-related effects on the outcomes.

## Conclusion

Our results show that there are significant changes in CT and CV after cataract surgery. The most pronounced changes occurred in the first postoperative month. The most probable causes are acute inflammatory processes, hemodynamic adjustments to postoperative IOP fluctuations and metabolic reactions to improved optical transmission. Further studies with longer follow-up periods, larger patient cohorts and standardized image analysis are required to clarify the clinical significance of these findings.

## Data Availability

The original contributions presented in the study are included in the article/Supplementary material, further inquiries can be directed to the corresponding authors.
