# Supplementary material for: Changes of individual choroidal thickness post-uneventful cataract surgery determined by spectral-domain optical coherence tomography over a 3-month period
Source: Front Med (Lausanne). 2026 Feb 18;13:1750805. doi: 10.3389/fmed.2026.1750805 (PMC12956627; doi:10.3389/fmed.2026.1750805)
Supplement: Supplementary file 1 [file Data_Sheet_1.pdf]

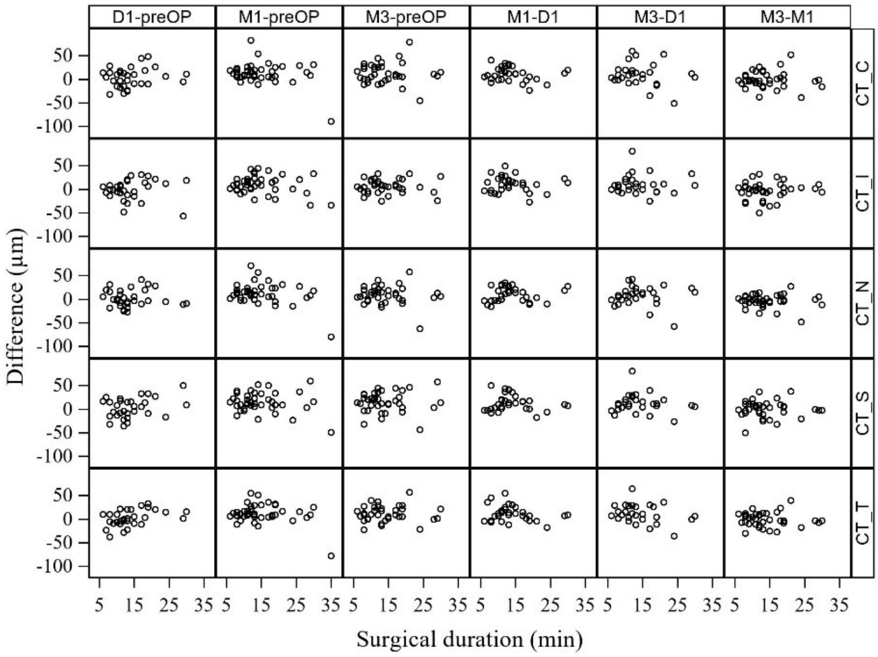

Supplementary Figure 1. *Scatter plots illustrating the relationship between surgical duration and postoperative changes in choroidal thickness (CT) between baseline and the respective follow-up time points*

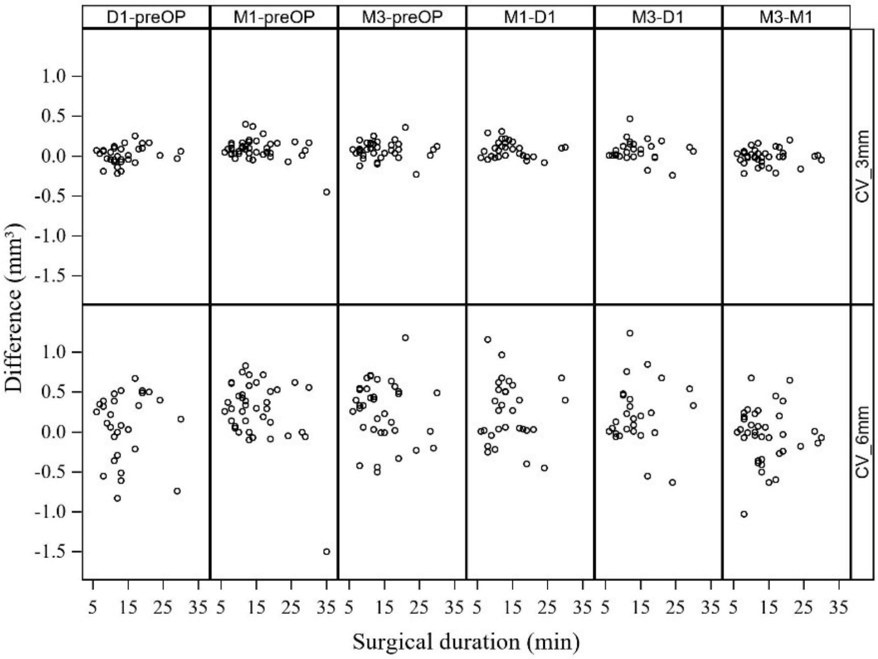

Supplementary Figure 2*. Scatter plots illustrating the relationship between surgical duration and postoperative changes in choroidal volume (CV) between baseline and the respective follow-up time points.*

Parameter / ETDRS grid	mean (SD) V1 N=5	mean (SD) V2 N=4	mean (SD) V3 N=5	mean (SD) V4 N=5
CT_C	219.40 (54.10)	235.25 (45.33)	234.80 (56.86)	233.80 (58.54)
CT_I	206.40 (51.68)	229.50 (62.05)	231.20 (66.01)	216.80 (57.45)
CT_N	190.00 (44.99)	202.25 (37.60)	206.00 (48.53)	198.60 (44.09)
CT_S	221.60 (68.21)	225.25 (65.09)	236.60 (69.19)	233.40 (62.52)
CT_T	220.60 (61.17)	231.00 (71.98)	233.40 (75.42)	230.20 (72.22)
CV__3mm	1.49 (0.39)	1.58 (0.40)	1.61 (0.45)	1.56 (0.41)
CV__6mm	5.55 (1.52)	5.83 (1.62)	5.93 (1.68)	5.76 (1.56)

Supplementary Table 1*. Descriptive statistics of choroidal thickness (CT) and choroidal volume (CV) in eyes developing cystoid macular edema (CME+) following uncomplicated cataract surgery. Mean values and standard deviations are shown for baseline (V1), postoperative day 1 (V2), month 1 (V3) and month 3 (V4).*
